# Changes in the body fluids proteome as a reflection of the physiological effects of dry immersion

**DOI:** 10.3389/fphys.2023.1178077

**Published:** 2023-04-24

**Authors:** L. K. Pastushkova, A. G. Goncharova, D. N. Kashirina, I. M. Larina

**Affiliations:** Institute of Biomedical Problems of the Russian Academy of Sciences, Moscow, Russia

**Keywords:** proteins, dry immersion, mass spectrometry, blood, urine

## Introduction

“Dry” immersion” (DI) as an experimental effect on the human body is the most adequate model of certain effects observed in space flight (SF). The time of development, the degree and severity of functional changes caused by DI are the closest to those occurring in the conditions of a real flight in low Earth orbit (Grigoriev, 1978; Kozlovskaya, 2008; Tomilovskaya, 2011). It is noted that DI causes a fast gravity-dependent response (Navasiolava et al., 2011; Tomilovskaya et al., 2019; Amirova et al., 2020). On the cardiovascular system, DI has a more powerful effect than the model with head-down bed rest −6° (HDBR) (Amirova et al., 2020). DI is accompanied by a more pronounced effect on body fluids than HDBR, with acute central hypervolemia in the first hours and subsequent hypovolemia (Navasiolava et al., 2011; Tomilovskaya et al., 2019). In a short time, within 3–7 days, a change in sensorimotor functions occurs: a decrease in the accuracy of movement control, a decrease in the accuracy of visual tracking, and an increase in sensitivity to vestibular signals. Review articles detail the physiological changes observed in DI studies in male volunteers (Pavy-Le Traon et al., 2007; Navasiolava et al., 2020; Watenpaugh, 2016; Tomilovskaya et al., 2019; Pandiarajan and Hargens, 2020) and female volunteers (Tomilovskaya et al., 2021).

## Blood and urine proteome in the study of response mechanisms to dry immersion conditions

**FIGURE 1:**
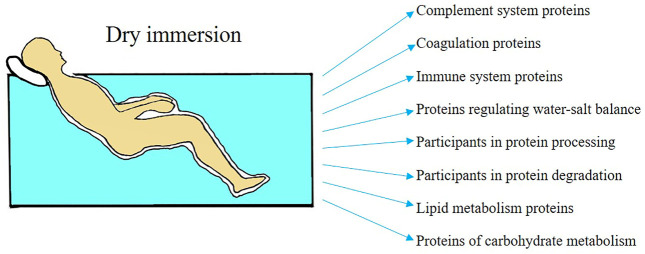
Influence of dry immersion factors on proteins involved in various biological processes.

The use of OMIC technologies to study the relationship between the known physiological effects of DI and the regulation of the functions of the main body systems is still limited. However, currently our team is actively investigating changes in the proteome of fluids (blood and urine) as a reflections of physiological effects and features of modification of molecular pathways and biological processes under DI conditions of various durations. Figure 1 presents generalized data on the effect of dry immersion factors on the blood proteome.

So, in a 5-day “dry” immersion, blood plasma samples of 14 healthy male participants of the experiment, aged 19–26 years, were analyzed. On the 2nd and 3rd days of the experiment, an increase in the peak area of fragments of a number of proteins - C3 and C4 of the complement system, high-molecular-weight kininogen and fibrinogen was found, which could be explained by the body's adaptation to the experimental conditions. On the 1st day of the recovery period, a significant increase in the peak areas of apolipoprotein CI and fragments of complement component C4 and fibrinogen was noted (Pastushkova et al., 2011).

In addition, the regulation of the main biological processes of homeostasis was reflected in the change in the presence of a number of proteins in the urine. Thus, 9 significantly changed proteins were identified: serum albumin (ALB), alpha-2-HS-glycoprotein (AHSG), cystatin-C (CST3), vitamin D-binding protein (GC), kininogen-1 (KNG1), kallikrein −1(KLK1), perlican, fetuin, A, E − cadherin. Obviously, changes in the levels of a number of proteins depended on kidney functions (selectivity of the glomerular barrier, processes of sodium and water reabsorption, regulation of renal hemodynamics) under conditions of 5-day DI. However, some proteins with significantly changed concentrations repeated the results of proteomic blood analysis, namely, the change in the concentration of kininogen, which is important for blood coagulation, was similar in urine and blood. Proteins that regulate endothelial function also attracted attention. Changes in the proteome were characterized by rapid development and recovery to the background level after the end of exposure (Pastushkova et al., 2012).

In the same study, for the first time, the interactions between the regulatory circuits in the circulatory system that maintain functions of the cardiovascular system at an optimal level were analyzed. To analyze regulatory interactions at all stages of DI, the principal component method was used. The functionality of each circuit was analyzed as a whole, which made it possible to reveal hidden patterns that are not revealed when analyzing individual variables. For the first time, the degree of synchronization and the timing of inclusion of the regulatory circuits of the circulatory system into the adaptation process were determined, which were assessed on the basis of an analysis of heart rate variability (nervous circuit of regulation), biochemical parameters of blood (humoral regulation circuit) and the proteomic profile of urine (metabolic regulation circuit). The results of assessing the directivity, as well as the response time of various regulatory circuits, turned out to be consistent (Rusanov et al., 2020).

Direct profiling of blood serum was used to analyze changes in the protein composition of samples of participants divided into 3 groups during a 7-day DI: the control group without countermeasures, the group with the use of mechanical stimulation and the group with low-frequency myostimulation as prophylactic agents. When analyzing the areas of MS peaks, it turned out that on the 7th day of exposure, the peaks of fibrinopeptide A, angiotensin II, the fragments of high molecular weight kininogen and complement component C3 were significantly reduced compared to pre-experimental values in all experimental series. Consequently, changes in the levels of these proteins were not corrected by the countermeasures used. On the 7th day of restoration, the areas of all changed peaks did not return to pre-experimental values. At the same time, the severity of changes had significant individual variability. The data obtained indicated incomplete restoration of the blood proteome within 7 days after DI. It was noted that the use of myostimulation and mechanical stimulation as countermeasures partially compensated for the adverse effect of 7-day DI on the parameters of the coagulation system (fibrinopeptide A) and lipid metabolism (apolipoprotein CIII that was increased in the group without countermeasures) (Pakharukova et al., 2011).

By the method of 2D electrophoresis, blood plasma samples of 5 healthy volunteers obtained 7 days before the start of exposure, on the 7th day of DI and on the 7th day after the end of the experiment were analyzed in the study of 7-day DI. On the 7th day of the readaptation period, a significant increase in the level of apolipoproteins A-I, A-IV and E was noted compared with the background and the 7th day of DI. At the same time, there was a decrease in the level of α-, β-fibrinogen, complement factor C4B, and serum amyloid P. The results of cluster analysis confirmed changes in the protein composition during the period of readaptation of the body to normal living conditions after the completion of immersion. These data, obtained by various proteomics methods, are highly informative, and the results are similar: in particular, the change in the proteomic regulation of the complement cascade and the coagulation cascade was confirmed both by mass spectrometry and in two-dimensional electrophoresis with quantitative spot scanning (Trifonova et al., 2010).

Continuing studies of the effect of DI duration on the blood proteomic composition, the features of the proteome were analyzed during 21-day DI (10 young men). It was noted that under DI conditions, the content of proteins involved in the regulation of protein processing, significantly changed, and pro-inflammatory activation of the immune system was also revealed. Activation of complement and coagulation cascades, activation of endothelial dysfunction, changes in the regulation of the humoral link of the immune system are shown. The data obtained may indicate an increased risk of hypercoagulability and thrombosis under conditions of prolonged immersion (Larina et al., 2020a), although so far no cases of thrombosis have been reported in experiments with DI.

The study of the effect of rapid blood redistribution during tilt tests in the 21-day DI unexpectedly gained relevance due to the occurrence of mild hemorrhagic syndrome in the study participants. Signs of the development of stagnant and orthostatic purpura were assessed in 10 healthy male volunteers during tilt tests. Pastosity and local petechial hemorrhages in the soft tissues of the legs and feet were noted. Proteomic analysis of the protein composition of blood samples by high performance liquid chromatography with tandem mass spectrometry was performed. From the group of proteins whose levels significantly differed in DI, those directly associated with vascular damage and changes in vascular permeability were isolated. It was shown that the levels of plasminogen, kallikrein, fibronectin, other coagulation and fibrinolysis factors changed; the complement system was activated. The data obtained are of practical importance as an attempt to stratify the risk of petechial hemorrhages and to evaluate new methods for the prevention the adverse effects of DI aimed at reducing reperfusion damage to soft tissues and parenchymal organs (Pastushkova et al., 2021).

For the first time, in order to clarify gender characteristics, a chromato-mass-spectrometric analysis of dry blood spots of 6 women participating in 3-day DI was carried out. It was found that the levels of proteins that regulate the processes of carbohydrate metabolism and proteasomal degradation of proteins are the first to change in response to the conditions of “dry” immersion. Then, on the second day, proteins of the immune response are involved and the level of an angiogenesis inhibitor, the pigment epithelium derived factor PEDF, decreases. By the third day, proteins that regulate the cascades of complement and coagulation, platelet degranulation, hemostasis, organization of the extracellular matrix, glycolysis, water-salt balance, and the antioxidant system were identified as reactants. It was noted that on day 3 of DI, the levels of histidine triad nucleotide-binding protein 1 (HINT1) and myosin light chain 4 (MYL4), which play an important role in the adaptation of the heart to volume overload, increased. Two days after the end of the experiment, only part of the proteins, whose levels responded to exposure, returned to the background level. At the same time, changes in the levels of proteins involved in lipid metabolism were noted as the most inert, occurring only after the completion of the experiment (Kashirina et al., 2022). We also note that there were no changes in the level of proteins associated with sex. However, the study of gender differences is planned as a separate line of work in the future.

Comparative analysis of the results of studying the proteome of biological fluids of the human body under the influence of space flight factors included a comparison of data from both real space flights (13 cosmonauts before and after long-term (169–199 days) flights on the ISS) and ground-based experiments. It has been shown that nine common proteins (A1BG, A2M, SERPINA1, SERPINA3, SERPING1, SERPINC1, HP, CFB, TF) are detected, the level of which changes after landing, as well as in DI and HDBR -6° experiments. Common signaling pathways and biological processes, such as regulation of the complement cascade and hemostasis, post-translational phosphorylation of proteins, and changes in the direction of protein metabolism processes, indicate the similarity of the pathogenesis of changes in these physiological systems in ground-based experimental models and real space flight (Brzhozovskiy et al., 2019).

## Conclusion

In conclusion, it should be noted that the use of proteomics methods based on mass spectrometry in gravitational physiology is a breakthrough direction that provides tangible advantages over previously accepted approaches. High sensitivity and accuracy are only part of the preference of proteomics over biochemical methods. Simultaneous, in one sample, obtaining data on the content of a large number of proteins serves as the basis for applying the results of bioinformatic programs to this array. The latter make it possible to move from the analysis of changes in individual proteins (both highly represented, and medium- and low-copy proteins) to the analysis of the direction of modifications of biological processes in the body of a healthy person. Moreover, proteomics methods make it possible to study the impact of extreme factors at the level of post-translational modifications of proteins (Larina, Brzhozovsky, et al., 2020c), as well as the mechanisms of the formation of protein-protein interaction networks in individuals adapted to SF factors (Larina, Kashirina, et al., 2020b).

Thus, studies of the proteome of blood and urine fluids under DI or HDBR conditions of various durations and prolonged space flights allow us to draw the following conclusions.

1) there is a pathogenetic correspondence between the selected experimental models and a complex of factors acting in a real space flight in relation to the development of effects in certain physiological systems;
2) changes in the proteome of body fluids develop in accordance with the timing of inclusion in the regulation circuits of molecular pathways of adaptation of the main biological processes;
3) changes in the proteome during DI - a reflection of physiological effects at all levels of homeostasis regulation: intracellular, cellular, tissue, organ, *etc.*
4) further studies of modifications of the proteome, which changes under the influence of a complex of simulated or real space flight factors, are promising for the development of directions for the prevention changes in the physiological systems of the body during deep space exploration and in practical medicine for the relief and control of conditions associated with forced hypokinesia of various durations.
